# Clinical correlates of early onset antipsychotic treatment resistance

**DOI:** 10.1177/02698811221132537

**Published:** 2022-10-21

**Authors:** Daniela Fonseca de Freitas, Deborah Agbedjro, Giouliana Kadra-Scalzo, Emma Francis, Isobel Ridler, Megan Pritchard, Hitesh Shetty, Aviv Segev, Cecilia Casetta, Sophie E. Smart, Anna Morris, Johnny Downs, Søren Rahn Christensen, Nikolaj Bak, Bruce J. Kinon, Daniel Stahl, Richard D. Hayes, James H. MacCabe

**Affiliations:** 1 Institute of Psychiatry, Psychology and Neuroscience, King’s College London, London, UK; 2 Department of Psychiatry, University of Oxford, Oxford, UK; 3 Division of Psychology and Language Sciences, University College London, London, UK; 4 South London and Maudsley NHS Foundation Trust, London, UK; 5 Norwich Medical School, University of East Anglia, Norwich, UK; 6 Sackler Faculty of Medicine, Tel Aviv University, Tel Aviv, Israel; 7 Shalvata Mental Health Center, Hod Hasharon, Israel; 8 Department of Health Sciences, Università degli Studi di Milano, Milan, Italy; 9 MRC Centre for Neuropsychiatric Genetics and Genomics, Cardiff University, Cardiff, UK; 10 H. Lundbeck A/S, Copenhagen, Denmark; 11 Lundbeck Pharmaceuticals LLC, Deerfield, IL, USA; 12 Cyclerion Therapeutics, Cambridge, MA, USA

**Keywords:** Psychotic disorders, schizophrenia, treatment refractory, antipsychotic agents, clozapine

## Abstract

**Background:**

There is evidence of heterogeneity within treatment-resistant schizophrenia (TRS), with some people not responding to antipsychotic treatment from illness onset and others becoming treatment-resistant after an initial response period. These groups may have different aetiologies.

**Aim:**

This study investigates sociodemographic and clinical correlates of early onset of TRS.

**Method:**

Employing a retrospective cohort design, we do a secondary analysis of data from a cohort of people with TRS attending the South London and Maudsley. Regression analyses were conducted to identify the correlates of the length of treatment to TRS. Predictors included the following: gender, age, ethnicity, problems with positive symptoms, problems with activities of daily living, psychiatric comorbidities, involuntary hospitalisation and treatment with long-acting injectable antipsychotics.

**Results:**

In a cohort of 164 people with TRS (60% were men), the median length of treatment to TRS was 3 years and 8 months. We observed no cut-off on the length of treatment until TRS presentation differentiating between early and late TRS (i.e. no bimodal distribution). Having mild to very severe problems with hallucinations and delusions at the treatment start was associated with earlier TRS (~19 months earlier). In sensitivity analyses, including only complete cases (subject to selection bias), treatment with a long-acting injectable antipsychotic was additionally associated with later TRS (~15 months later).

**Conclusion:**

Our findings do not support a clear separation between early and late TRS but rather a continuum of the length of treatment before TRS onset. Having mild to very severe problems with positive symptoms at treatment start predicts earlier onset of TRS.

## Introduction

Treatment-resistant schizophrenia (TRS) has been defined as the absence of a satisfactory response to treatment with at least two different antipsychotics with adequate dose and duration (Howes et al., 2017). A recent meta-analysis of first-episode psychosis (FEP) studies showed that 24% of patients with schizophrenia became treatment-resistant (Siskind et al., 2022). Most people who develop TRS (70%–84%) show treatment resistance from illness onset, while a smaller proportion develops TRS after a period of treatment response (Ajnakina et al., 2020; Correll et al., 2019; Demjaha et al., 2017; Kinon, 2019; Lally et al., 2016). In the Aetiology and Ethnicity in Schizophrenia and Other Psychoses study, people with FEP were followed up for 10 years and the observed mean length of treatment before the development of late TRS was 5 years (Demjaha et al., 2017).

The observed heterogeneity in TRS raises questions about the aetiology of treatment resistance and there is evidence to support multiple neurobiological pathways to TRS (Potkin et al., 2020). It has been suggested that early TRS may represent a neurobiologically distinct subtype of schizophrenia, associated with abnormalities in glutamate transmission but normal dopamine function (Kinon, 2019; Potkin et al., 2020). In contrast, late TRS is suggested to result from iatrogenic supersensitivity of dopamine receptors due to prolonged dopamine blockade treatment by high-potency antipsychotic treatment (Kinon, 2019; Potkin et al., 2020). If early and late treatment resistance are neurobiologically distinct, they may have different clinical and demographic correlates, which could improve our understanding of mechanistic and aetiological differences between these groups.

Few studies have investigated the sociodemographic and clinical factors that differentiate between early and late TRS (Ajnakina et al., 2020; Correll et al., 2019; Lally et al., 2016). Lally et al. (2016) and Ajnakina et al. (2020) report the findings of a study with 80 patients identified with early TRS or late TRS, where the latter was defined as treatment resistance after an initial period of symptomatic remission for at least 6 months. Gender differences were observed: men were more likely to show early TRS (Lally et al., 2016). No group differences were observed in the duration of untreated psychosis, age, ethnicity, IQ, alcohol or cannabis consumption, or negative symptoms (using the Positive and Negative Syndrome Scale (PANSS)) (Lally et al., 2016). However, comparing only early and late TRS, it can be observed that the severity of positive symptoms was higher in the group with early TRS (Ajnakina et al., 2020). In a survey asking psychiatrists about their patients with TRS, Correll et al. (2019) report that patients who developed TRS within the first 5 years of treatment were younger at illness onset than those who showed TRS later.

### Aim of the study

The study aimed to investigate sociodemographic and clinical correlates of early TRS. We hypothesised that our findings would corroborate the previous literature regarding associations with gender and age, namely that male gender, younger age at treatment onset and greater severity of positive symptoms at treatment onset would be associated with early TRS.

## Methods

### Setting

Using a retrospective cohort study design, we do a secondary analysis of data from a TRS cohort. We used data from South London and Maudsley National Health Service Foundation Trust (SLaM) electronic health records (EHRs). SLaM provides mental healthcare to four South London boroughs (Southwark, Lewisham, Lambeth and Croydon), with a population of 1.3 million people (Stewart et al., 2009). Information on EHRs records, both structured and free-text fields, was accessed using the Clinical Record Interactive Search (CRIS), a system that was established, within robust governance, after the implementation of the EHRs in 2006 (Perera et al., 2016; Stewart et al., 2009). At the time of the data extraction, CRIS provided access to the de-identified information of over 330,000 individuals. Natural Language Processing algorithms are used to retrieve information from the free-text fields. These allow clinical data (e.g. prescription of medication) to be extracted with high precision and recall, outperforming a simple keyword search (Jackson et al., 2017; Perera et al., 2016). CRIS has been approved for secondary data analysis by the Oxford C Research Ethics Committee (18/SC/0372). All projects using CRIS are submitted to an oversight committee led by service-users (Perera et al., 2016; Stewart et al., 2009).

### Sample inclusion criteria

SLaM service-users who (i) had a primary diagnosis of a schizophrenia spectrum disorder (ICD-10: F20–F29), (ii) were prescribed antipsychotics between 1 January 2007 and 31 December 2017, and (iii) lived within the SLaM catchment area, or were of no fixed abode, at the time of the prescription of the first antipsychotic after 1 January 2007, were eligible for inclusion in the sample. Since we did not have data prior to 2007, it was not possible to determine the date of the first prescription. Consequently, we excluded individuals where treatment resistance was observed within 3 months from the record of the first antipsychotic after 1 January 2007, as these were likely to be historical cases.

### Ascertainment of treatment resistance

Treatment resistance was coded manually, as it ensured the assessment of the non-adequate response to antipsychotics was as accurate as possible. This process took approximately 1 h per participant, on average. A random sample of individuals (10%) who met the inclusion criteria (i)–(iii) was manually coded to ascertain the existence of treatment resistance (Kadra-Scalzo et al., 2022). Following the Treatment Response and Resistance in Psychosis Working Group guidelines (Howes et al., 2017), TRS was defined as a failure to respond to two different antipsychotics (⩾6-week trials each). Failure to respond was assumed when a switch was made to a new antipsychotic where the reason for the switch was explicitly due to non-response and/or when the reason was not related to adverse side effects or non-adherence with treatment. Furthermore, cases were considered to have TRS if they were treated with clozapine. For the TRS individuals, the date of TRS was defined as the earliest that either of the following two criteria for TRS was met: the date of the initiation of the third antipsychotic (after failure to respond to two trials) or the first treatment with clozapine (Kadra-Scalzo et al., 2022).

### Potential correlates of the length of treatment before the onset of TRS

The outcome was the length of treatment (in days) between the prescription of the first antipsychotic after 1 January 2007 to the TRS date. The exposures fell into three categories: sociodemographic characteristics, clinical factors and potential markers of non-compliance.

Sociodemographic information included gender, age at the first antipsychotic treatment (between 2007 and 2017) and ethnicity. Ethnicity was grouped into White (British, Irish and other White backgrounds), Black (African, Caribbean, White and Black African, White and Black Caribbean and any Other Black background) and Other ethnicities (Bangladeshi, Chinese, Indian, Pakistani, White and Asian, any Other Asian background, any Other Mixed background, any Other ethnic group or ethnicity not stated).

Information on psychiatric diagnoses was retrieved from 1 January 2007 to the TRS date. Where an individual had multiple diagnoses within the schizophrenia spectrum, we used a hierarchy: the diagnosis of schizoaffective disorder (ICD-10: F25) prevailed over other diagnoses, and schizophrenia (ICD-10: F20) prevailed over other psychotic disorders (ICD-10: F21–F24, F28–F29). Psychiatric co-morbidities were grouped in disorders related to substance use (ICD-10: F10–F14, F16, F18–F19), mood (ICD-10: F30–39, F42.1), anxiety (ICD-10: F40–F43), personality (ICD-10: F60–F61) and development (ICD-10: F70–F79, F80–F84, F88, F90).

The severity of symptoms, at the time of the first antipsychotic prescription in the observation window, was assessed using two items from the Health of the Nation Outcome Scale (HoNOS): (i) problems associated with hallucinations and delusions and (ii) problems with activities of daily living (Mukherjee and Sazhin, 2022; Wing et al., 1998). Given the multiple assessments, we used a hierarchy: first, we retrieved the HoNOS scores in the 3 months before the first antipsychotic prescription; if none were available, we searched for information in the 3 months after the first antipsychotic prescription; if this was also unavailable, we retrieved the most recent HoNOS scores before the first antipsychotic prescription. The ratings were dichotomised into ‘minor or no problem’ (original scores 0–1) and ‘mild to very severe problem’ (original scores 2–4) (Mansour et al., 2020).

We used two proxies for medical non-compliance within the observation window: being involuntarily hospitalised and being treated with a long-acting injection (LAI) antipsychotic. Compulsory hospitalisation included only medical detentions under Part 2 (i.e. non-forensic) of the Mental Health Act 1983 (MHA, HM Government, 1983).

### Statistical analyses

We used multiple ordinary least squares regression to investigate crude and adjusted associations between the outlined sociodemographic and clinical factors and length of treatment between the first antipsychotic and TRS date. We checked the assumptions of the regression analyses, namely the normality and homoscedasticity of residuals. To impute missing data, we used multivariate imputation using chained equations (MICE) with the assumption of missing at random data (Sterne et al., 2009; White et al., 2011). Regressions using multiple imputations allow a more efficient and less biased estimation of model parameters than using a complete case analysis. The imputation model did not include any auxiliary variables other than the variables of the substantive model. Based on guidance, the number of datasets to be created of imputed data using MICE was equal to, or higher, than the percentage of incomplete cases (White et al., 2011). The regression estimates presented here (crude and adjusted) are pooled based on the datasets of imputed data using MICE. As a sensitivity analysis, we performed a complete case analysis to ensure that the large missingness of HoNOS and model misspecification did not influence our results. Complete case analysis also gives valid results if the probability of being a complete case is independent of the outcome given the covariates (but not the outcome) in the model at the cost of loss of precision (Héraud-Bousquet et al., 2012). All analyses were conducted in STATA 15 (StataCorp, 2017).

## Results

### Participants

**Figure 1.**
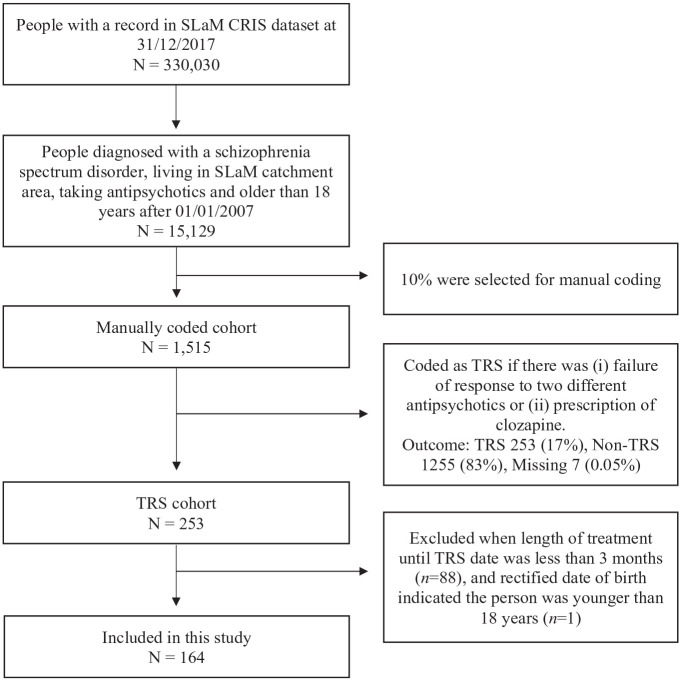
Selection of study cohort.

From a dataset of 1515 cases that were manually coded, there were 253 service-users who met the inclusion criteria and were identified as TRS; 1255 individuals who were rated as treatment responsive were excluded, and seven were excluded because of missing data and were not able to be coded (Kadra-Scalzo et al., 2022). Of the 253 TRS cases, 88 presented treatment resistance within less than 3 months of the first antipsychotic prescription after 1 January 2007, and one was observed to be younger than 18 at the time of the first antipsychotic; these cases were excluded from the analyses (Figure 1). The remaining sample of 164 cases in the study was predominantly men (60.4%) and service-users of a Black ethnicity (54.9%). The median age was 37 years. A co-morbid substance use disorder was diagnosed in 34% of the cohort. The majority of service-users had been involuntarily hospitalised (62.8%) and had been treated with a long-acting injectable antipsychotic (58.5%) before meeting the criteria for TRS. The total percentage of missing data in the dataset was low (1.33%), but missingness in the two HoNOS items was relatively high (14.0% in HoNOS hallucinations, 14.6% in HoNOS daily activities). So, for 14.6% of cases with incomplete data, we created 20 datasets of imputed data (White et al., 2011). Missing data were predicted only by gender; women were less likely to have missing data in HoNOS (odds ratio = 0.20, 95% confidence interval (CI): 0.05, 0.69). See Table 1 for further descriptive information and Supplemental Table S1 for descriptive statistics among people with missing data and no-missing data.

**Table 1.** Descriptive statistics of the length of treatment until TRS and cohort characteristics.

	*n* (%), Total *N* = 164
Outcome	
Length of treatment to TRS (days)	Md = 1338, IQR: 636 – 2183, range: 91–3905
Sociodemographic	
Age (years)	Md = 37.3; IQR: 27.9 – 47.9, range: 18.4 – 78.5
Gender – male (R)	99 (60.4)
Ethnicity	
White (R)	55 (33.5)
Black	90 (54.9)
Other	19 (11.6)
Psychiatric diagnosis	
Schizophrenia spectrum diagnosis	
Schizophrenia	108 (65.9)
Schizoaffective	31 (18.9)
Other chronic psychosis	25 (15.2)
Co-morbidities	
Any substance use	25 (15.2)
Mood disorders	56 (34.2)
Anxiety-related disorders	21 (12.8)
Personality disorder	29 (17.7)
Developmental disabilities	16 (9.8)
Symptomatic severity (HoNOS items)*	
Hallucinations and delusions, problem of mild or high severity (14.0% missing data)	84 (59.6)
Activities of daily living, problem of mild or high severity (14.6% missing data)	46 (32.9)
Service use possible related to medical non-compliance	
Involuntary hospitalisation (MHA Part 2)	103 (62.8)
LAI antipsychotic	96 (58.5)

* HoNOS items were calculated from available data. There were no missing data in other variables other than HoNOS.

HoNOS: Health of the Nation Outcome Scale; IQR: interquartile range; LAI: long-acting injection; Md: median; MHA: Mental Health Act; TRS: treatment-resistant schizophrenia.

### Length of treatment before TRS and its correlates

**Figure 2.**
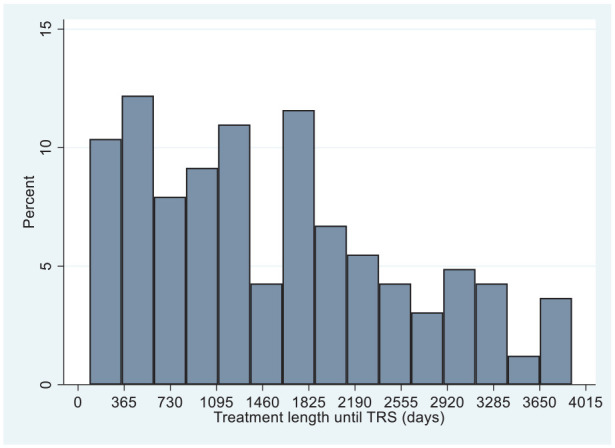
Distribution of length of treatment until TRS onset. TRS: treatment-resistant schizophrenia.

The median interval between the first treatment and TRS was 3 years and 8 months (Table 1). The incidence of TRS was uniform across the duration of treatment, with no evidence of a bimodal distribution. Thus, we decided to use the length of treatment in days instead of creating a binary variable of early/late TRS (Figure 2).

The univariable regression analyses revealed that earlier TRS was associated with the main diagnosis; compared to schizophrenia (ICD-10 F20), people diagnosed with other chronic psychosis (ICD-10: F21–F24, F28–F29) had TRS earlier, while people with a schizoaffective disorder (ICD-10: F25) had TRS later (Table 2). In addition, in the univariable regression analyses, earlier TRS was associated with the following: having mild to very severe problems with hallucinations at the time of treatment start, having mild to very severe problems with activities of daily living at the time of treatment start, not having been involuntary hospitalised and not having received antipsychotic treatment via an LAI. However, in the fully adjusted model, only the intensity of hallucinations and delusions at the time of the first antipsychotic was associated with an earlier onset of TRS (on average, 583.78 days earlier, 95% CI: 248.22–919.34, *p* = 0.001, i.e. more than one and a half years earlier). No significant associations were observed regarding gender and age at the treatment start (Table 2).

**Table 2.** Sociodemographic and clinical factors associated with treatment length until presentation of treatment resistance.

	Crude regression coefficients; *B* [95% CI]*	Adjusted regression coefficients *B* [ 95% CI]*
Sociodemographic		
Age (years)	−3.61 [−15.30, 8.09], *p* = 0.543	−1.60 [−10.09, 13.29], *p* = 0.787
Female gender	−153.85 [−474.39, 166.69], *p* = 0.345	−22.04 [−345.93, 301.85], *p* = 0.893
Ethnicity: White (R)		
Black	289.89 [−51.17, 630.95], *p* = 0.095	264.56 [−80.39, 609.51], *p* = 0.132
Other ethnicities	−129.34 [−659.63, 400.94], *p* = 0.631	79.75 [−424.92, 584.42], *p* = 0.755
Psychiatric diagnosis		
Schizophrenia spectrum diagnosis: Schizophrenia (R)		
Schizoaffective	515.81 [120.42, 911.20], *p* = 0.011	334.15 [−66.36, 734.66], *p* = 0.101
Other chronic psychosis	−440.96 [−871.64, −10.28], *p* = 0.045	−206.36 [−657.31, 244.58], *p* = 0.367
Co-morbidities		
Any substance use	242.39 [−193.39, 678.17], *p* = 0.274	380.01 [−50.46, 810.47], *p* = 0.083
Mood disorders	274.05 [−54.76, 602.86], *p* = 0.102	−28.25 [−364.32, 307.82], *p* = 0.868
Anxiety related disorders	203.79 [−265.66, 673.25], *p* = 0.393	182.79 [−302.98, 668.57], *p* = 0.458
Personality disorder	260.78 [−149.32, 670.88], *p* = 0.211	53.21 [−370.92, 477.34], *p* = 0.805
Developmental disabilities	13.39 [−516.48, 543.25], *p* = 0.960	93.31 [−438.91, 625.53], *p* = 0.729
Symptomatic severity (HoNOS items)*		
Hallucinations and delusions, problem of mild or high severity	−581.31 [−913.35, −249.28], *p* = 0.001	−583.78 [−919.34, −248.22], *p* = 0.001
Activities of daily living, problem of mild or high severity	−379.48 [−737.94, −21.01], *p* = 0.038	−223.53 [−579.94, 132.88], *p* = 0.216
Service use possibly related to medical non-compliance		
Involuntary hospitalisation (MHA Part 2)	689.94 [382.80, 997.11], *p* < 0.001	287.75 [−77.98, 653.48], *p* *=* 0.122
LAI antipsychotic	610.34 [305.56, 915.10], *p* < 0.001	276.41 [−67.44, 620.25], *p* *=* 0.114

* Ordinary least squares regression coefficients were pooled across 20 multiply imputed datasets from 164 observations. R = reference category in regression analyses.

CI: confidence interval; HoNOS: Health of the Nation Outcome Scale; LAI: long-acting injection; MHA: Mental Health Act.

In the sensitivity analyses, using only the 140 complete cases, similar regression coefficients were observed (see Supplemental Table S2). The only exception was that treatment with an LAI remained as a predictor of later TRS in the adjusted model (and not only in the unadjusted model as observed in the analyses with multiple imputation). In the complete case analysis, people who received an LAI were identified with TRS later than those who were not treated with an LAI (on average, 459.82 days (i.e., 1 year and 3 months) later, 95% CI: 94.05, 825.59, *p* = 0.014; see Supplemental Table S2). In the analyses with multiple imputation, LAI was associated with later TRS (on average 276.41 days later (i.e., 9 months), 95% CI: −67.44, 620.25, *p* = 0.114), but the CIs showed the association was not significant.

## Discussion

This study aimed to identify the sociodemographic and clinical predictors of early treatment resistance in a sample of people with TRS. After adjusting for clinical and demographic factors, only the severity of problems with positive symptoms at the time of the first antipsychotic prescription was related to an earlier presentation of TRS. On a complete case analysis, which is subject to selection bias, treatment with an LAI antipsychotic was also associated with later TRS. Contrary to our hypotheses, neither gender nor age at the treatment start was associated with the length of treatment until the onset of TRS.

There is one main difference between our study and previous research on early/late TRS (Ajnakina et al., 2020; Correll et al., 2019; Demjaha et al., 2017; Lally et al., 2016). Previous studies focused on investigating predictors of early, or late, onset of TRS compared to the non-development of TRS; while our study focused exclusively on exploring if there are sociodemographic and clinical factors related to the length of treatment in a cohort of cases of TRS. Our data analysis on the length of treatment to TRS showed no bimodal distribution. Therefore, after the initial inspection of the outcome, we investigated the correlates of treatment length to TRS as a continuum rather than a dichotomous category.

The observation of a constant incidence of TRS over a wide observation window suggests that there may not be a clear-cut categorical distinction between TRS subgroups, at least not one that can be detected only with reference to the length of treatment. While heterogeneity in response is recognised, the observed continuum in the length of time to antipsychotic non-response may suggest that there can be multiple neurobiological pathways to TRS, and TRS may be more related to the accumulation of multiple risk factors over time than one single neurobiological risk factor (Kinon, 2019; Potkin et al., 2020).

Only having mild to very severe problems with hallucinations and delusions, that is the severity of psychotic symptoms, at first antipsychotic prescription was associated with a shorter length of time to treatment resistance. Data from a previous study (Ajnakina et al., 2020) showed people with TRS from the first treatment had more positive symptoms (using PANSS) than people whose TRS occurred after a period of remission of symptoms. This finding is also consistent with the observation that people with TRS who were hospitalised during FEP had TRS earlier than those who were not hospitalised during FEP (Kanahara et al., 2018), although these differences were marginally non-significant (*p* = 0.050).

In addition, there is some evidence to suggest that people treated with LAI antipsychotics had a longer duration of treatment before the identification of TRS. This was only observed in the complete case analyses, which could be subject to selection bias. As LAIs are used when there are concerns of compliance (Barnes et al., 2020), people who are perceived as more cooperative (and were not treated with LAI) may be prescribed clozapine earlier (which was one criterion for our ascertainment of TRS). The improved bioavailability of LAI antipsychotics, combined with fewer opportunities for non-adherence, may result in more stable and higher true plasma concentrations than their equivalent in oral medications (McEvoy, 2006). Furthermore, until recently, the majority of LAIs were first-generation, high-potency antipsychotics (Stone et al., 2018). Thus, the association of LAIs with late TRS, seen in the sensitivity analysis, is in line with the hypothesis that late TRS arises through dopamine supersensitivity following sustained high-dose, high-potency antipsychotic treatment (Chouinard et al., 2017). Given that this finding can be subject to bias, further research should investigate whether LAIs are associated with the length of treatment before TRS in other cohorts. Furthermore, researchers should investigate if this potential association is related to the management of non-compliance and delays in identifying TRS, other pathological processes or if it results from dopamine supersensitivity.

No association between sociodemographic characteristics and length of treatment before TRS was observed. Our research does not corroborate previous findings on the association between early TRS and male gender (Ajnakina et al., 2020; Lally et al., 2016) and younger age at illness onset (Correll et al., 2019). Again, methodological differences, namely having two distinct groups of treatment length (i.e. early vs late TRS), could explain the observed differences. However, given the scarcity of studies on the subject, further investigation is needed to gain solid knowledge about the sociodemographic predictors of longer antipsychotic treatment before treatment resistance onset.

### Strengths and limitations

A major study strength is its sampling methods. First, the sample comprises service-users attending a free mental healthcare service that provides services to a geographically defined population. Hence, the sample is representative of the SLaM catchment area, and there is almost no selection bias. Second, the TRS definition applied follows the recommended criteria for treatment resistance (Howes et al., 2017), namely in the number and duration of antipsychotic trials. However, we were unable to include the dose of medication in our definition of TRS. Another limitation of the study is that some clinical factors, namely psychiatric comorbidity, were measured at any time before TRS and not exclusively at illness onset; thus, people who showed treatment resistance after a longer duration of treatment will have a longer time of follow-up, and this could affect the availability of information regarding psychiatric diagnosis and service use (i.e., surveillance bias). Given that electronic records were only fully established near 2007 (Stewart et al., 2009), we may not have precise dates for the first antipsychotic treatment of people who entered the cohort in the first years of the observation window. Furthermore, the fact that we used information readily available in the clinical records meant we could not assess the severity of the psychotic illness using standard scales (e.g. PANSS), nor include information on other potential predictors, such as the duration of untreated psychosis. Our operationalisation of TRS depended on clinicians’ assessment of what an adequate response should be and their decision to change medication when such was not observed.

## Conclusions

We employed a data-driven approach to studying potential subtypes of TRS in a manually coded gold-standard dataset. The presence of problems with psychotic symptoms at treatment start (from mild to very severe problems) was associated with earlier TRS. In a sensitivity analysis including only complete cases and subject to selection bias, treatment with LAIs was associated with later TRS. Further research is needed to develop solid knowledge about factors that can explain the heterogeneity in antipsychotic non-response.

## Supplemental Material

sj-docx-1-jop-10.1177_02698811221132537 – Supplemental material for Clinical correlates of early onset antipsychotic treatment resistance Supplemental material, sj-docx-1-jop-10.1177_02698811221132537 for Clinical correlates of early onset antipsychotic treatment resistance by Daniela Fonseca de Freitas, Deborah Agbedjro, Giouliana Kadra-Scalzo, Emma Francis, Isobel Ridler, Megan Pritchard, Hitesh Shetty, Aviv Segev, Cecilia Casetta, Sophie E. Smart, Anna Morris, Johnny Downs, Søren Rahn Christensen, Nikolaj Bak, Bruce J. Kinon, Daniel Stahl, Richard D. Hayes and James H. MacCabe in Journal of Psychopharmacology
