# Supplementary material for: Clinical correlates of early onset antipsychotic treatment resistance
Source: J Psychopharmacol. 2022 Oct 21;36(11):1226–33. doi: 10.1177/02698811221132537 (PMC9643817; doi:10.1177/02698811221132537)
Supplement: sj-docx-1-jop-10.1177_02698811221132537 – Supplemental material for Clinical correlates of early onset antipsychotic treatment resistance [file sj-docx-1-jop-10.1177_02698811221132537.docx]

**Supplementary material**

**Table S1.** *Descriptive statistics of the length of treatment until TRS and cohort characteristics among people with missing data and no-missing data*

|  | ***Sample with no-missing data , n* = 140** | ***Sub-sample with missing data on HoNOS*, n* = 24** | ***p-value ^a^*** |
| --- | --- | --- | --- |
| ***Outcome*** |  |  |  |
| *Length of treatment to TRS (days)* | *Md* = 1527, *IQR*: 616 – 2183 | *Md* = 1629, *IQR*: 519 – 2219 | *p* = 0.825 |
| ***Sociodemographic*** |  |  |  |
| Age (years) | *Md* = 38.8; *IQR*: 27.9 – 47.9 | *Md* = 36.8; *IQR*: 27.9 – 47.9 | *p* = 0.825 |
| Gender - Male | 56.4% | 83.3% | *p* = 0.013 |
| Ethnicity |  |  | *p* = 0.694 |
| White | 33.6% | 33.3% |  |
| Black | 55.7% | 50.0% |  |
| Other | 10.7% | 16.7% |  |
| ***Psychiatric diagnosis*** |  |  |  |
| Schizophrenia spectrum diagnosis |  |  | *p* = 0.800 |
| Schizophrenia | 65.0% | 71.8% |  |
| Schizoaffective | 20.0% | 13.5% |  |
| Other chronic psychosis | 15.0% | 16.7% |  |
| Comorbidities |  |  |  |
| Any substance use | 16.4% | 8.3% | *p* = 0.537 |
| Mood disorders | 34.3% | 33.3% | *p* = 1.000 |
| Anxiety-related disorders | 12.9% | 12.5% | *p* = 1.000 |
| Personality disorder | 17.1% | 20.8% | *p* = 0.772 |
| Developmental disabilities | 8.6% | 16.7% | *p* = 1.000 |
| ***Symptomatic severity (HoNOS items)**** |  |  |  |
| Hallucinations and delusions, problem of mild or high severity | 60.0% | N/A^b^ |  |
| Activities of daily living, problem of mild or high severity | 32.9% | N/A^b^ |  |
| ***Service use possible related to medical non-compliance*** |  |  |  |
| Involuntary hospitalisation (MHA Part 2) | 64.3% | 54.2% | *p* = 0.367 |
| Long-acting injection antipsychotic | 60.7% | 45.8% | *p* = 0.185 |
| Notes: ^a^ *p*-values were calculated using Fisher’s exact test or the k-sample equality-of-medians test. ^b^ In the group with missing data, only one person had data on the HoNOS hallucinations and delusions; no one had information on the HoNOS activities of daily living. | | | |

**Table S2.** *Sensitivity analyses including only complete cases*

|  | **B [95% CI]*** |  | **Adjusted B[ 95% CI]*** |
| --- | --- | --- | --- |
| ***Sociodemographic*** |  |  |  |
| Age (years) | -2.64 [-15.21, 9.93], *p* = .679 |  | -1.84 [-10.54, 14.22], *p* = .769 |
| Female gender | -98.76 [-443.22, 245.70], *p* = .572 |  | 30.21 [-292.26, 352.67], *p* = .853 |
| Ethnicity: White (R) |  |  |  |
| Black | 304.55 [-65.12, 674.21], *p* = .106 |  | 306.79 [-51.22, 664.80], *p* = .092 |
| Other ethnicities | -128.58 [-722.25, 465.10], *p* = .669 |  | 121.00 [-543.42, 785.43], *p* = .719 |
| ***Psychiatric diagnosis*** |  |  |  |
| Schizophrenia spectrum diagnosis: Schizophrenia (R) |  |  |  |
| Schizoaffective | 532.41 [112.56, 952.27], *p* = .013 |  | 348.407 [-121.29, 818.10], *p* = .145 |
| Other chronic psychosis | -463.23 [-933.56, 7.10], *p* = .054 |  | -203.16 [-666.92, 260.60], *p* = .388 |
| Comorbidities |  |  |  |
| Any substance use | 151.20 [-309.59, 611.99], *p* = .518 |  | 334.60 [-97.27, 766.46], *p* = .128 |
| Mood disorders | 206.54 [-152.04, 565.11], *p* = .257 |  | -50.71 [-404.04, 302.62], *p* = .777 |
| Anxiety related disorders | 278.47 [-230.24, 787.18], *p* = .281 |  | 265.49 [-195.55, 726.53], *p* = .257 |
| Personality disorder | 311.10 [-139.59, 761.79], *p* = .175 |  | 85.83 [-379.90, 551.57], *p* = .716 |
| Developmental disabilities | 152.55 [-457.75, 762.85], *p* = .622 |  | 127.98 [-417.06, 673.02], *p* = .643 |
| ***Symptomatic severity (HoNOS items)**** |  |  |  |
| Hallucinations and delusions, problem of mild or high severity | -591.91 [-926.44, -257.38], *p* = .001 |  | -581.01 [-919.35, -242.67], *p* = .001 |
| Activities of daily living, problem of mild or high severity | -379.48 [-737.90, -21.06], *p* = .038 |  | -209.84 [-521.36, 101.67], *p* = .185 |
| ***Service use possibly related to medical non-compliance*** |  |  |  |
| Involuntary hospitalisation (MHA Part 2) | 679.41 [341.35, 1017.47], *p* < .001 |  | 176.84 [-179.03, 532.72], *p =* .327 |
| Long-acting injection antipsychotic | 741.11 [413.95, 1068.27], *p* < .001 |  | 459.82 [94.05, 825.59], *p =* .014 |
| * Coefficients are based on 140 complete cases. | | | |
